# Compact Multilayer Film Structure for Angle Insensitive Color Filtering

**DOI:** 10.1038/srep09285

**Published:** 2015-03-19

**Authors:** Chenying Yang, Weidong Shen, Yueguang Zhang, Kan Li, Xu Fang, Xing Zhang, Xu Liu

**Affiliations:** 1 State key laboratory of Modern Optical Instrumentation, Department of Optical Engineering, Zhejiang University, Hangzhou 310027, China

## Abstract

Here we report a compact multilayer film structure for angle robust color filtering, which is verified by theoretical calculations and experiment results. The introduction of the amorphous silicon in the proposed unsymmetrical resonant cavity greatly reduces the angular sensitivity of the filters, which is confirmed by the analysis of the phase shift within the structure. The temperature of the substrate during the deposition is expressly investigated to obtain the best optical performance with high peak reflectance and good angle insensitive color filtering by compromising the refractive index of dielectric layer and the surface roughness of the multilayer film. And the outlayer of the structure, worked as the anti-reflection layer, have an enormous impact on the filtering performance. This method, described in this paper, can have enormous potential for diverse applications in display, colorful decoration, anti-counterfeiting and so forth.

Thin film have been widely used in a variety of fields, e.g., display, sensing, communication, spectral analysis, and solar cells^1^. A great deal of researches focused on the design and manufacture of the specific multilayer thin film for various purpose, such as beam splitter, bandpass filters, blocking filters, chirped mirrors, FTR filters and so forth^2^. Color filter, which is a common kind of bandpass filter, displays various colors by selectively transmitting or reflecting a specific wavelength in the visible region. In recent years, color filters have been extensively studied for its wide range of application, e.g., display^3^,^4^,^5^,^6^, colorful decoration^7^, and anti-counterfeiting^8^,^9^,^10^. Generally, color filter can be grouped into two categories in terms of the principle of the color filtering feature: optical color filter and chemical color filter. Traditional chemical color filter comprising dye or pigment changes the color of the reflected light as the result of a wavelength-selective absorption of the particular functional groups and has a relatively high angular tolerance. However, those constituent chemical dye or pigments are not stable to a wide range of processing chemicals, cannot stand long-time illumination with strong light intensities, require lots of processes for filter patterns, and cause a significant environmental burden simultaneously. On the other hand, guided mode resonance (GMR) filter, a kind of the optical filters, has a sub nanometer bandwidth with very high efficiency and color filters in reflection with high purity can be obtained. But its optical property is high angle sensitive and the angular tolerance is quite poor^11^,^12^.

Benefited from the tremendous advance in numerical simulation algorithms and nano/micro fabrication techniques, nanostructured color filters combined with structural color have been investigated extensively and developed in different aspects for the past few years^13^,^14^,^15^. Nevertheless, it is disappointed that the angular tolerance of these color filters are not considered in most of the works, which means a shift of spectrum with different incidence angles. For many applications, e.g., special illumination, display and spectral analysis, the same perceived specular color of the filters is required for a broad range of incidence angles. Therefore, it is highly desired to improve the incident angular tolerance of the color filters. In 2013, Yi-Kuei Ryan Wu proposed a new scheme through the localized resonance in metallic nanoslits by light funneling, in which angle insensitive color filters have been achieved with the capability of tuning a wide color across the entire visible band^16^. However, only TM polarization of the incident light was considered, restricted by the underlayer of one dimensional grating and cone of the incident light characterized by the azimuthal angle was neglected, which limits its further application in many fields. Besides, the fabrication of such filter required complicated procedures with multiple steps of nanoimprint lithography, reactive ion etching, and e-beam evaporation, which limits their potential application in large area circumstance. Given these reasons, thin film devices are investigated as the alternative method. Tae-Hui Noh^17^ and L. Jay Guo^18^ demonstrated that the high refractive index material such as TiO_2_ and Si_3_N_4_ can be employed as the spacing dielectric layer to reduce the dependence of the viewing angle for the Fabry-Perot cavity-based spectral filter. Meanwhile, Mikhail A. Kats proposed a scheme using ultra-thin highly absorbing film on a metallic substrate to produce various colors with a low sensitivity to the incidence angle^19^. This year, Jae Yong Lee presented a colorful organic hybrid solar cell structure with ultra-thin amorphous silicon, which can transmit various colors. In this structure, the ultra-thin amorphous silicon layer induces a negligible propagating phase change, as well as an extraordinary reflection phase change on the interface with the metal layer to achieve the constructive light interference, which leads to high angular tolerance of the transmitted colors^20^. However, owing to the inevitable absorption in ultrathin Ag and silicon layer, the reported peak transmittance is limited to 30% and the saturation of the proposed colors is not so good with an improvable angular tolerance. Furthermore, the reflection color filter has a wider application in colorful decoration, package printing, anti-counterfeiting and e-book display.

Here we propose a new approach to achieve the angle robust color filtering for reflection with convenient process of simple multilayer film deposition, which is available for large area manufacture. Three primary RGB (Red, Green, Blue) colors are produced with high saturation and brightness. The significantly improved angular property is achieved by adopting the material of high refractive index instead of common oxides in the proposed unsymmetrical resonant cavity, which can highly confine the phase shift at the interface and in the dielectric layer. The temperature of the deposition is expressly investigated to obtain the best optical performance, via balancing the refractive index of dielectric layer and the surface roughness of the multilayer.

## Results

### Multilayer-film-based angle insensitive color filtering

**Figure 1:**
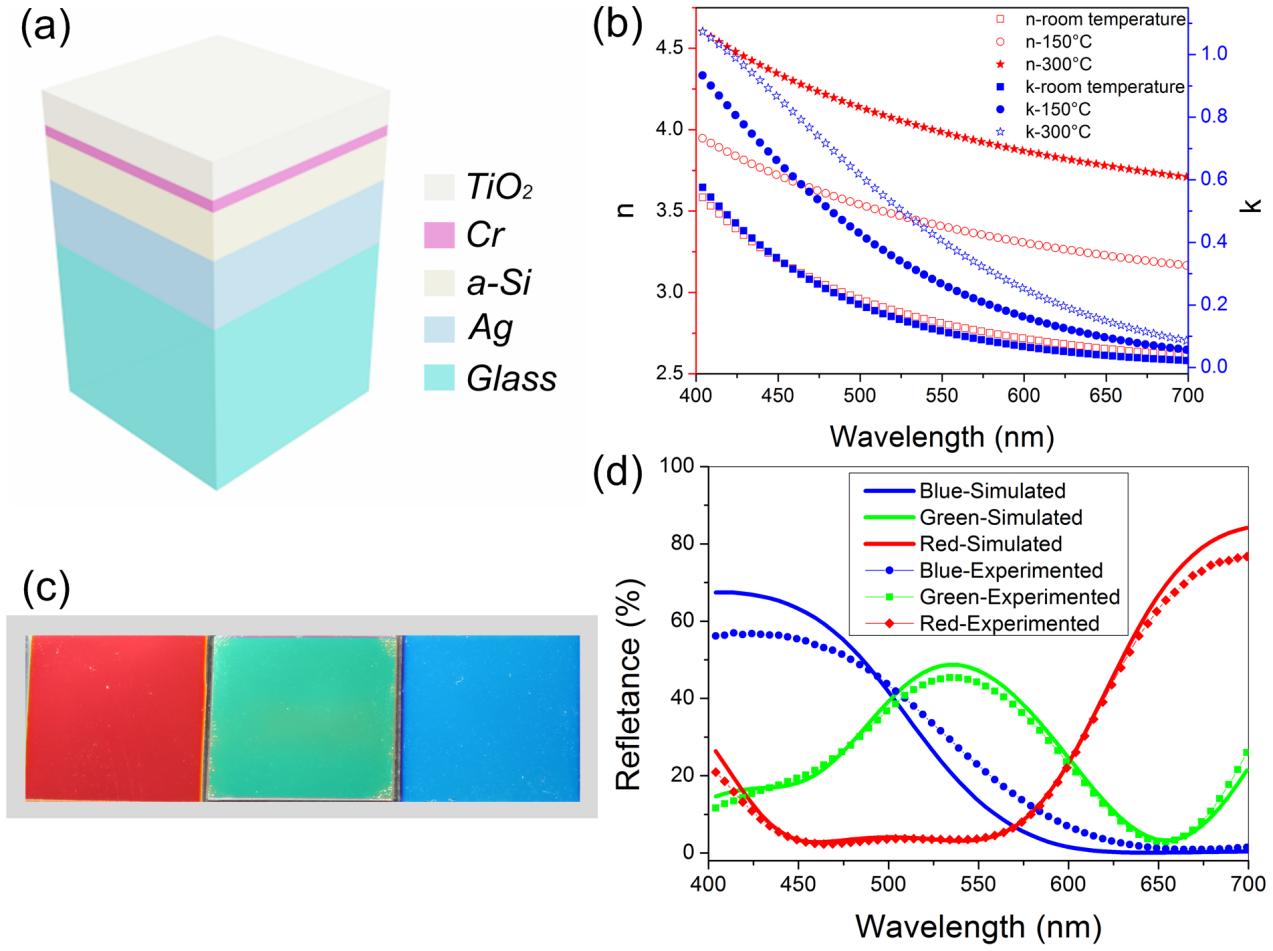
(a) The schematic diagram of the proposed reflective angle robust color filtering device. (b) The complex refractive indices of the amorphous silicon film deposited by e-beam evaporation with the substrate at room temperature, 150°C, and 300°C. (c) A photograph of the fabricated RGB devices at normal incidence. (d) The simulated and experimental reflection spectra of the proposed color filters for the three colors of red, green, and red color at normal incidence with the unpolarized light.

The schematic geometry of the proposed color filter is shown in Fig. 1(a). It is an unsymmetrical resonant cavity comprised of an amorphous silicon (a-Si) dielectric film sandwiched by a layer of thin chromium (Cr) film and a layer of thick silver (Ag) film with thickness larger than 100 nm. In our work, silver is selected as the material of the bottom metal because of its lowest material absorption loss and highest reflectivity over all the visible wavelengths among all metals. To achieve the best color saturation, Chromium is adopted as a partial reflection mirror and the absorptive layer due to its intrinsic property of n/k≈1, i.e., the refractive index is approximately equal to its extinction coefficient. The amorphous silicon layer is a phase matching layer (also called spacer layer) whose optical thickness determines the wavelength of peak reflectance. The outer titanium dioxide (TiO_2_) film is an effective anti-reflection layer for chrome film to reduce the reflection further and improve the color saturation. The two light beams reflected respectively from the a-Si/Ag interface and the Air/chrome interface will form the interference constructively at the peak wavelength while the destructive interference result in a low reflection at the other wavelength of the visible region and the light is absorbed by the chrome layer. With this design, a brilliant residual color in reflection can be observed.

In device fabrication process, firstly, the bottom Ag was deposited by thermal evaporation on the clean substrate of fused silica at room temperature. The thickness of the bottom Ag layer should be larger than 100 nm to block the transmitted light. It is noted that the substrate should be heated and kept at 150°C before the following steps, which would greatly improve the angular performance of the device because of a much higher refractive index of amorphous silicon. With the substrate kept at 150°C, the a-Si, Cr, and TiO_2_ layers were deposited sequentially by e-beam evaporation. The complex index of the a-Si was derived by the spectrometry method considering both the reflectance and the transmittance curves^21^. The study of the complex index of a-Si was emphasized in the aspect of the deposition temperature, displayed in Fig. 1(b). It can be seen that both the real part n and the imaginary part k increases with a rising temperature. Theoretically, a relatively high temperature is beneficial for the angular tolerance, illustrated in the paragraph below. The thickness of these three layers a-Si, Cr, and TiO_2_ are all taken into account as the design parameters to produce the RGB colors, shown in the Table 1. The detailed design rule of the thickness can be found in Supplementary Information. Though the thickness of these three layers are different for each color, the lift-off technique can be applied to receive the RGB color pixels on the same substrate^22^. Figure 1(c) shows a photograph of the fabricated reflective RGB filters taken at normal incidence. The simulated results calculated by transfer matrix method well match with the experimental results measured by spectrophotometer (OLYMPUS USPM-RU) at normal incidence, as shown in Fig. 1(d). Apparently, a slight distinction of the reflectance spectrum can be observed, specifically, the experimental peak reflectance is always a little lower than the simulated one, which can be attributed to the scattering loss resulted from the uneven surface of each layers.

**Figure 2:**
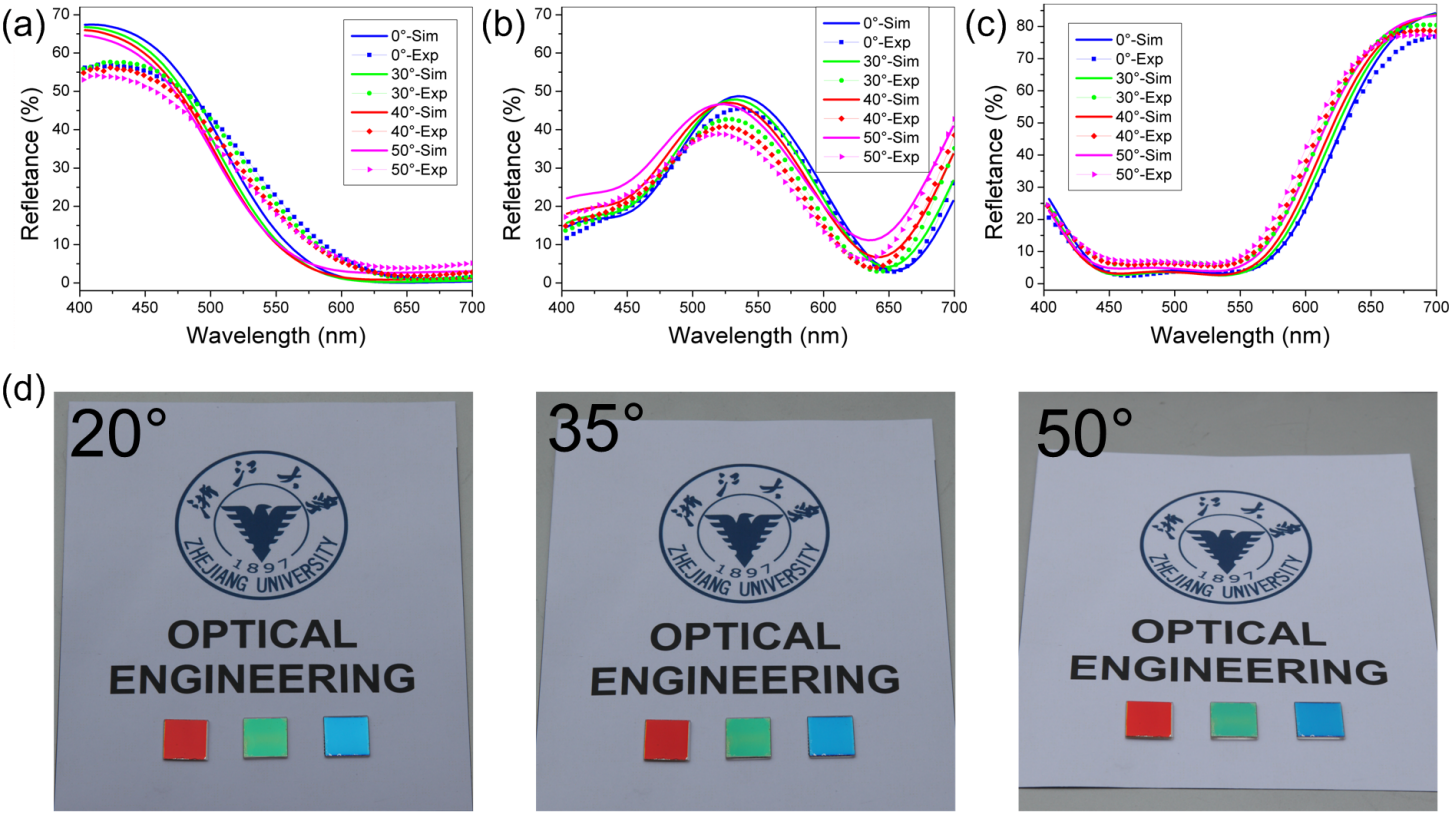
(a–c) The measured and calculated reflection spectra of the proposed RGB devices from the normal incidence to the oblique incidence of 30°, 40° and 50° under unpolarized light. (a) The blue color. (b) The green color. (c) The red color. (d) The optical images of the fabricated RGB filters taken with outdoor ambient light under sunshine at oblique incidence of 20°, 35°, 50°. The size of the fabricated color devices is 1.5 cm × 1.5 cm.

Good behavior of angle robust color filtering for the RGB color filters could be obtained with the designed parameters. The angle resolved reflection spectra of these three color filters for unpolarized incidence are displayed in Fig. 2. Obviously, the experimental results at the oblique incidence validates the simulated results as same as the normal incident case. The reflectance spectrums almost keep invariant with a small angle of incidence. As the incidence angle increases gradually, the peak reflectance wavelengths mainly remain the same and the peak of the reflection decreases whereas the reflection at blocking region rises, resulting in a wider bandwidth. On the basis of these measured reflectance spectrums, the chromaticity coordinates at various angles of incidence are calculated and marked in Figure S4. The images of these devices in Fig. 2(d) were taken with outdoor ambient light under sunshine at three different angles up to 50° and they show no color variations. The size of the fabricated color devices is 1.5 cm × 1.5 cm. It can be easily extended to large area manufacturing with flexible substrates, potentially useful in the fields of display, colorful decoration, and anti-counterfeiting.

### Physical origin for angle insensitive color filtering

**Figure 3:**
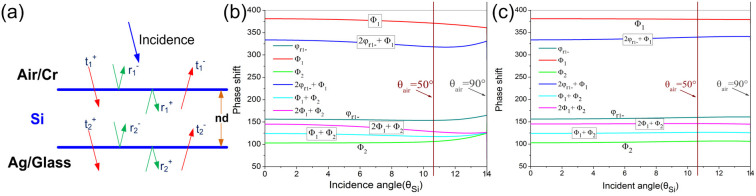
(a) The simplified model of the proposed angle robust color filter with multiple reflections and transmissions for the analysis of the phase shift within the structure. (b–c) The aggregate components of the phase shift arose in the derivation of the magnitude of the reflection for p-polarization (b) and s-polarization (c).

The phase shift generated within the proposed multilayer structure is studied thoroughly to reveal the physical origin of the angle robust spectral filtering feature of the present filter. In the analysis, the reflectance can be calculated by Smith method, also known as the Effective Interface method^23^. In the simplified model shown in Fig. 3(a), considering the intermediate a-Si layer, the total Fresnel reflection coefficient can be:


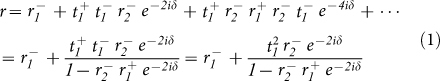
Then the intensity of the reflection can be derived:
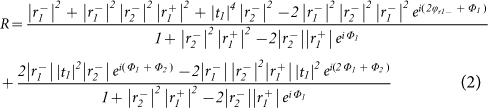

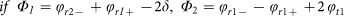
where φ_r1-_, φ_r2-_, and φ_r1+_, are the reflection phase shift on the interface between the corresponding medium while φ_t1_ is the transmission phase shift on the interface between a-Si and Cr. The propagation phase shift in the dielectric layer of a-Si is represented by δ, δ = −2πndcos(θ)/λ. Therefore, the magnitude of the reflection varies with the phase shift within the structure depending on the angle of incidence θ. As shown in equation (2), the reflectance expression is complicated, but the reflectance is mainly affected by the four phase items (*Φ*_1_, *Φ*_1_ + *Φ*_2_, 2*Φ*_1_ + *Φ*_2_, 2φ_r1-_ + *Φ*_1_) about the propagation phase shift in the silicon and the reflection/transmission phase shift on the interface. If the phase items keep invariable with the incidence angle, the reflectance will remain the same and leads to the angle insensitivity properties. The assumption is verified by the simulation results shown in Fig. 3(b–c), which display the respective phase shift items in the realistic air/Cr/a-Si/Ag/glass structure for blue filter at λ = 409 nm as a function of incidence angle. The phase shift items including the calculation method are described in detail in Supplementary Information. Profit from large contrast of the refractive index between the incident medium of air and the modulating layer of a-Si, the critical angle of total reflection in the a-Si layer becomes very small (θ_critical_ = 14°), implying a specially limited angle of propagating across the crucial layer of a-Si. So, with such a small angle variations in the spacer layer, all the phase items will keep the constant up to 50 degrees whether at p-polarized incidence (as shown in Fig. 3(b)) or at s-polarized incidence (as shown in Fig. 3(c)), which results in the angle insensitivity of the color filter. With a large incidence angle over 50°, the phase shift will change gradually, which is attributed to the variation of dcos(θ)/dθ. It is obvious that the aggregate phase shift at s-polarized incidence behaves a little better than the p-polarization because of a higher effective admittance ratio between a-Si and air for the s-polarization light^2^. It is noted that the upper anti-reflection layer of TiO_2_ is left out here to simplify the analytical model of the phase shift and the above analysis is unaffected on this simplification.

### Effect of deposition temperature and TiO_2_ outlayer

**Figure 4:**
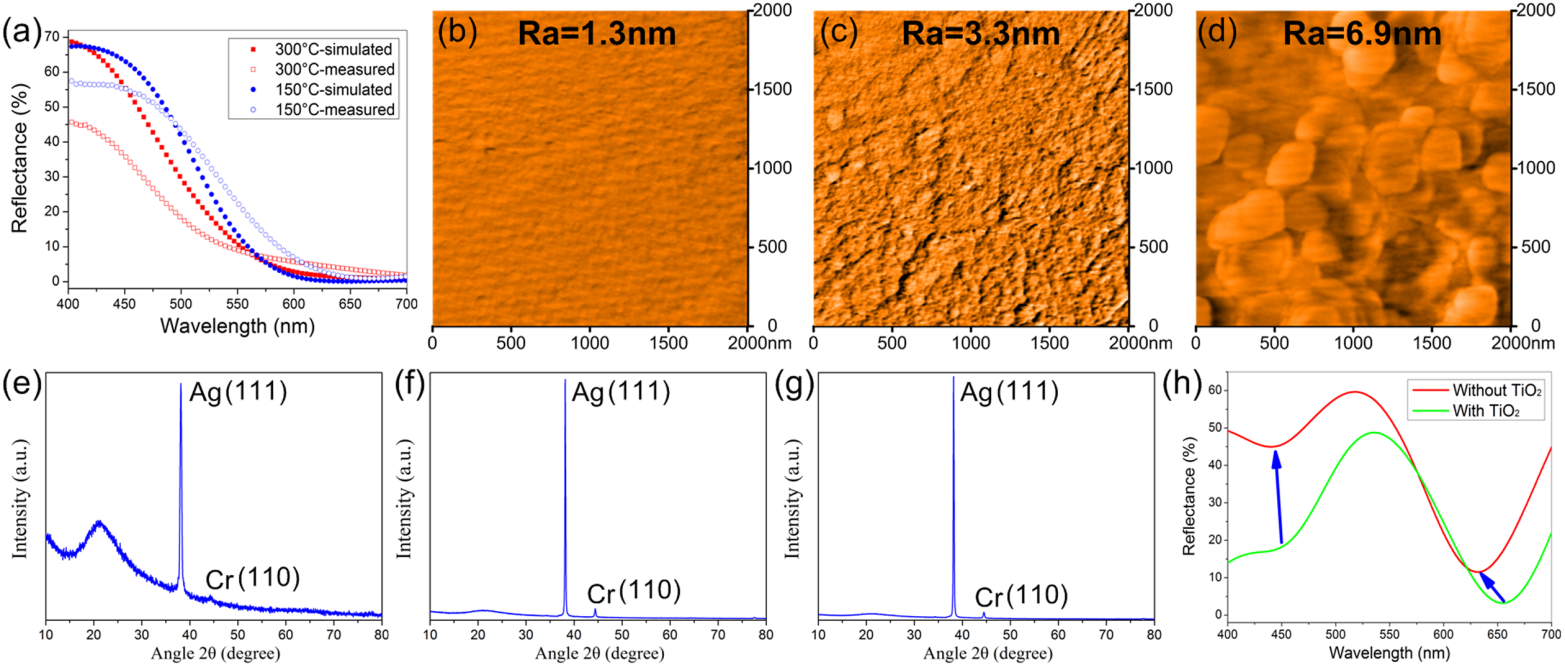
(a) The measured and calculated reflection spectra of the proposed Blue color device at normal incidence with the deposition temperature of 150°C and 300°C. (b–d) The AFM figures of the filters fabricated at different temperature. (b) Room temperature. (c) 150°C. (d) 300°C. (e–g) The XRD results of the filters fabricated at different temperature corresponding to the AFM results above. (e) Room temperature. (f) 150°C. (g) 300°C. (h) The reflectance spectra of the green filter with TiO_2_ and without TiO_2_.

On the basis of the discussion above, it is not difficult for us to draw a conclusion: the higher refractive index of the intermediate dielectric layer the better the angular tolerance. Thus, to increase the refractive index of the a-Si layer has a great influence on the optical property of the proposed devices. Empirically, the deposition temperature has a great impact on the refractive index of the deposited film. The higher deposition temperature can lead to a higher packing density with a larger refractive index as a result of the higher mobility of the particle forming film on the surface of the substrate^2^,^24^, as shown in Fig. 1(b). Thereby, we may choose 300°C or an even higher temperature for the deposition. Unfortunately, an elevated substrate temperature will cause the underlayer silver film to crystallize and increase the surface roughness of the fabricated devices, which will sorely degrade the performance of the fabricated devices. As shown in Fig. 4(a), the blue filter fabricated at 300°C presents a much lower peak reflectance than the simulation. Obvious scattering light can be observed during the reflectance measurement by OLYMPUS USPM-RU spectrometer. The AFM results in Figs. 4(b–d) show the surface topography of the devices deposited at different temperatures and explain the decrease of the reflectance for the device fabricated at 300°C. It can be observed that the size of the grains as well as the surface roughness increases explicitly with respect to deposition temperature. The average roughness of the samples for a 2 × 2 μm^2^ area are 1.3 nm and 3.3 nm with the substrate at room temperature and 150°C respectively, while the average roughness arises up to 6.9 nm at 300°C, which is confirmed by the large agglomerated clusters in Fig. 4(d). As well, the X-Ray Diffraction measurement was performed to further explore the intrinsic nature of the increased roughness, shown in Figs. 4(e–g). All the three devices corresponding to different deposition temperature show a characteristic Ag (1 1 1) diffraction peak and a Cr (1 1 0) diffraction peak, which demonstrates the crystallinity of Ag and Cr in the film. Apparently, by the comparison with the constant crystallinity of the fused silica substrate, the device fabricated at 300°C has the highest degree of crystallinity of silver material among the three devices, which further confirms the analysis of the AFM results above. Therefore, the temperature of the substrate during the deposition must be controlled to avoid the high crystallization of the silver film. To compromise the refractive index of a-Si layer and the surface roughness of the multilayer film, the deposition temperature in our experiment is set at 150°C to obtain the best optical performance.

It is worthy to mention that the upper TiO_2_ layer plays an important role in efficient color filtering. It acts as a broadband anti-reflection coating in the visible region. Without the anti-reflection layer, the performance deteriorates acutely with unspecified reflectance profile or much broad bandwidth appearing, illustrated in Fig. 4(h). Hence, an appropriate anti-reflection layer is of necessity to achieve the efficient color filtering behavior.

## Discussion

In conclusion, a novel method to produce the angle robust spectral filtering is proposed and verified experimentally. All the three fabricated primary RGB devices maintain the same perceived specular colors for a broad range of incidence angles with the average polarization, consistent with the simulated results. The scheme of phase shift within the proposed structure makes the origin of the angle robust color filtering clear, which is mostly ascribed to the small angle propagating across the layers caused by the high refractive index of a-Si. What is more, the upper anti-reflection layer helps to improve the color saturation significantly. Consequently, these angle robust reflective color filter has great potential in various applications such as display, colorful decoration, anti-counterfeiting and so forth.

## Methods

### Simulation

Simulation of the angle resolved reflectance spectra at normal and oblique incidence was performed by the transfer matrix method. The complex refractive index of amorphous silicon used in the simulation was calculated by spectrometry method considering both the reflectance and the transmittance curves.

### Device fabrication

The proposed filters were manufactured on the clean substrates of fused silica. The bottom layer of Ag with 100 nm was deposited by thermal evaporation with a vacuum pressure less than 2 × 10^−3^ Pa at room temperature. Prior to the deposition of subsequent thin films, the substrates were heated to 150°C. Then, with the substrate kept at 150°C, the a-Si, Cr, and TiO_2_ with different thicknesses for producing a specific color were successively deposited on the bottom Ag by e-beam evaporation.

### Optical characterization

The reflection spectrum of the fabricated devices at normal incidence was measured by a reflection spectrometer (OLYMPUS USPM-RU). The angle resolved reflectance was measured by the angle resolved spectrometer from Ideaoptics Company. The transmittance measurement was performed by a spectrophotometer (Shimadzu UV-3101PC). The measurement of the surface topography of the fabricated devices at different temperature was performed by atomic force microscopy (AFM-IIa). The crystallization of the films was carried out by X-ray diffractometer (Empyrean 200895, PANalytical B.V.).

## Supplementary Material

Supplementary Information Supplementary Information

## Figures and Tables

**Table 1:** Thickness of the constituent layers for the RGB filters

Color	RED	GREEN	BLUE
Ag	>100 nm	>100 nm	>100 nm
a-Si	82 nm	124 nm	35 nm
Cr	8 nm	7 nm	22 nm
TiO_2_	46 nm	21 nm	65 nm
