# Supplementary material for: Compact Multilayer Film Structure for Angle Insensitive Color Filtering
Source: Sci Rep. 2015 Mar 19;5:9285. doi: 10.1038/srep09285 (PMC5380129; doi:10.1038/srep09285)
Supplement: Supplementary Information [file srep09285-s1.pdf]

<Supplementary Information>

Compact Multilayer Film Structure for Angle Insensitive Color Filtering

Chenying Yang, Weidong Shen*, Yueguang Zhang, Kan Li, Xu Fang, Xing Zhang, and Xu Liu

* E-mail: adongszju@hotmail.com

State key laboratory of Modern Optical Instrumentation,
Department of Optical Engineering, Zhejiang University, Hangzhou 310027, China

The design rules to obtain the thickness of the film stack

In the proposed structure, as shown in Fig. S1, the thick silver is chosen to provide high reflectivity while ultra-thin chrome with the intrinsic property n≈k is selected as a partial reflection mirror and the absorptive layer. The amorphous silicon layer is a phase matching layer to induce the highest reflectance at specific wavelength whose optical thickness determines the wavelength of peak reflectance. The outer TiO_2 film is an effective anti-reflection layer for chrome film to reduce the reflection further and improve the color saturation.

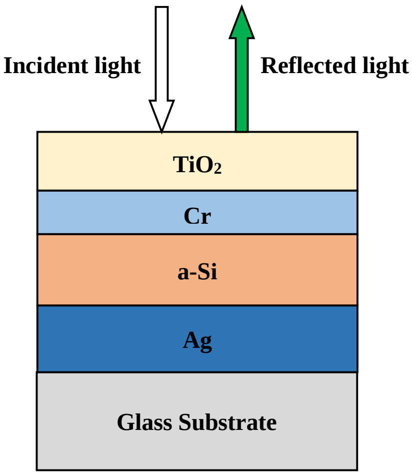

Figure S1 | Schematic of the proposed reflective color filter

In the simplified model of Air|Cr|a-Si|Ag|Glass, the refractive index of the incident medium and the exit medium are n_0 and n_s while the optical constant and the thickness of Cr, a-Si, and Ag are set as follow in Table S1:

Table S1 | The optical constant and the thickness of Cr, a-Si, and Ag

**Layer**	**Optical constant**	**Thickness**
Cr	n-ik	d
a-Si	n_1-ik_1	D
Ag	n'-ik'	>100nm

The absorption of Cr is so large that no light can transmit through the Cr layer with the thickness up to 40nm. Therefore, an ultrathin Cr film with $d \ll \lambda_0$ is employed and the phase thickness of Cr film is $\delta = \frac{2\pi}{\lambda}(n-ik)d$. The characteristic matrix of Cr film is

$$M = \begin{bmatrix} \cos\delta & i\sin\delta/(n-ik) \\ i(n-ik)\sin\delta & \cos\delta \end{bmatrix} \quad \text{(S1)}$$

The thickness d is much smaller than the wavelength ($\frac{d}{\lambda} \to 0$), therefore δ approaches to zero and cosδ, sinδ can be expanded by Taylor's series. The matrix M can be thereby simplified by ignoring high order components as shown in the right part of Eq. (S2):

$$M \approx \begin{bmatrix} 1 & i2\pi d / \lambda \\ 4\pi nkd / \lambda & 1 \end{bmatrix} \qquad \text{(S2)}$$

Suppose the effective admittance of film stack a-Si|Ag|Glass is Z=X+iY and the characteristic matrix of film stack a-Si|Ag|Glass is

$$M' = \begin{bmatrix} B' \\ C' \end{bmatrix} \quad \text{where} \quad \mathrm{Z} = \mathrm{X} + \mathrm{iY} = C'/B' \qquad \text{(S3)}$$

Therefore, the equivalent admittance of film stack Cr|a-Si|Ag|Glass is

$$\eta = \frac{4\pi nkd / \lambda + X + iY}{1 - 2\pi dY / \lambda + i2\pi dX / \lambda} \qquad \text{(S4)}$$

The reflectance of the whole structure Air|Cr|a-Si|Ag|Glass can be calculated:

$$R = \left(\frac{n_s - \eta}{n_s + \eta}\right)\left(\frac{n_s - \eta}{n_s + \eta}\right)^* = \frac{(n_s - X - \frac{2\pi}{\lambda} dYn_s - \frac{4\pi}{\lambda} knd)^2 + (\frac{2\pi}{\lambda} dXn_s - Y)^2}{(n_s + X - \frac{2\pi}{\lambda} dYn_s + \frac{4\pi}{\lambda} knd)^2 + (\frac{2\pi}{\lambda} dXn_s + Y)^2} \qquad \text{(S5)}$$

From equation (S5), it is not hard to be found: if X→∞ and Y→∞, R→1 can be obtained. The admittance is the so-called matching admittance for highest reflection.

So an appropriate thickness of a-Si can induce highest reflection at specific wavelength. The thickness of a-Si at specific wavelength λ_0 can be determined by equation (S5). The a-Si layer can be divided into two separate layers for different functions. The bottom a-Si layer contacting with Ag is to transform the admittance to zero and the optical thickness of the top a-Si layer should be odd times of $\lambda_0/4$ to obtain the so-called matching admittance for highest reflection.

Suppose the thickness of the top a-Si layer contacting with Ag and the bottom a-Si layer contacting with Cr are D_1 and D_2, respectively. Thus, $D_2 = (2m+1)\lambda_0 / 4n_1$ (m is an integer). The thickness of Ag layer is larger than 100nm and can be treated as the substrate in thin film optics. At the approximation of n'≈0, $D_1 = \frac{\lambda_0}{2\pi n_1}\arctan(\frac{k'}{n_1})$ can be obtained without consideration of extinction coefficient of a-Si. So the thickness of a-Si can be reached

$$D = \frac{\lambda_0}{2\pi n_1}\arctan(\frac{k^{'}}{n_1}) + (2m+1)\lambda_0 / 4n_1 \qquad \text{(S6)}$$

where λ_0 is the desired highest reflection wavelength and m is the interference order.

Based on this principle, the structure is optimized and designed for RGB colors. Taking an example of the green filter, λ_0=520nm, n_1=3.48, k'=3.32, m=1, the thickness of a-Si is calculated, D=130nm. The reflectance is shown in Figure S2 with the calculated thickness of a-Si. The peak reflectance wavelength shows a shift about 15nm from the desired wavelength, which can be attributed to the approximation of n'≈0 and n≈k, and the neglect of the absorption of a-Si. Here the interference order m=1is chosen to receive a proper bandwidth.

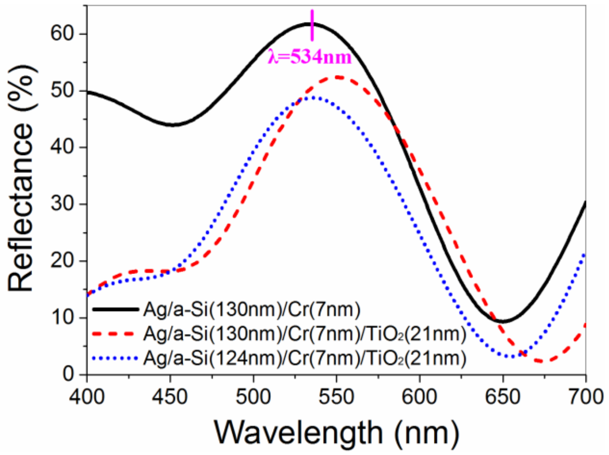

Figure S2 | The reflectance of the film stack with the calculated thickness of a-Si for green color and the optimized result.

In addition, the thickness of TiO_2 is determined by $\lambda'/4n_{TiO2}$ to realize the anti-reflection at the blocking wavelength region, where λ' is the specific wavelength in the blocking region and n_{TiO2} is the refractive index of TiO_2. The thickness of chrome is no more than 30nm to pass the visible light into the following film stack to obtain the desired reflection.

In short, the design rule is an effective way to get the rough thickness of every layer. Based on these initial parameters, the properties of the color filter can be further optimized to obtain the accurate thickness of each layer using commercial optical coating software.

The phase shift in the film stack

From the reflectance equation (2) in the paper, we could find that the reflectance is mainly affected by the reflection phase shift φ_{r1-}, φ_{r2-}, and φ_{r1+}, the transmission phase shift φ_{t1} and the propagation phase shift δ. If the phase shift items keep invariable with the incidence angle, the reflectance will remain the same, leading to the angle insensitivity feature. The propagation phase shift in the a-Si layer is calculated by $\delta=-2\pi nd\cos(\theta)/\lambda$. The angle of refraction in the a-Si layer is small, less than 14 °, because of the large contrast of the refractive indexes between the air and the silicon. Therefore, the propagation phase shift in the a-Si layer is nearly invariable, due to the negligible variation of $\cos(\theta)$ from 1 to 0.983 with the angle in silicon layer from 0 °to 10.69 °corresponding to the angle in the air from 0 °to 50 °. The transfer matrix method is employed to calculate the reflection/transmission phase shift on the interface. Taking the example of φ_{r1+} for p polarization, it is the reflection phase shift generated on the interface of a-Si/Cr. In the calculation, film stack a-Si|Cr|Air is considered, shown in the Figure S3. The characteristic matrix of Cr|Air is

$$M=\begin{bmatrix}1\\Y\end{bmatrix}B=\begin{bmatrix}\cos\delta_1 & \mathbf{i}\sin\delta_1/\eta_1\\ \mathbf{i}\eta_1\sin\delta_1 & \cos\delta_1\end{bmatrix}\begin{bmatrix}1\\ \eta_{Air}\end{bmatrix}$$

where $\delta_1=2\pi n_1 d_1\cos(\theta_1)/\lambda$, $\eta_1=n_1/\cos(\theta_1)$ for p polarization, $\eta_{Air}=n_{Air}/\cos(\theta_{Air})$, and Y is the equivalent admittance of the film stack Cr|Air. Thus, the reflection coefficient r and the reflection phase shift φ_{r1+} can be calculated by $r=(\eta_{a-Si}-Y)/(\eta_{a-Si}+Y)=r_1+\mathbf{i}r_2, \varphi_{r1+}=\tan^{-1}(r_2/r_1)$. When the incidence angle in the a-Si layer varies from 0 °to 10.69 °, $\cos(\theta_1)$ almost keeps unchanged, which results in the elements in the characteristic matrix nearly invariable. In addition, the admittance of a-Si η_{a-Si} is also kept. So the reflection phase shift remains the same as the angular insensitivity required. Other reflection/transmission phase shift can be calculated in the same way. So the angle insensitivity of these reflection/transmission phase shift mainly results from the high refractive index of amorphous silicon.

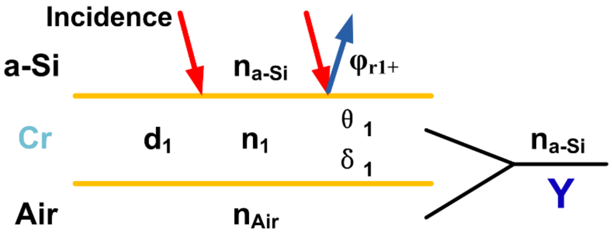

Figure S3 | The schematic diagram of the film stack a-Si|Cr|Air for the calculation of the reflection phase shift φ_{r1+}.

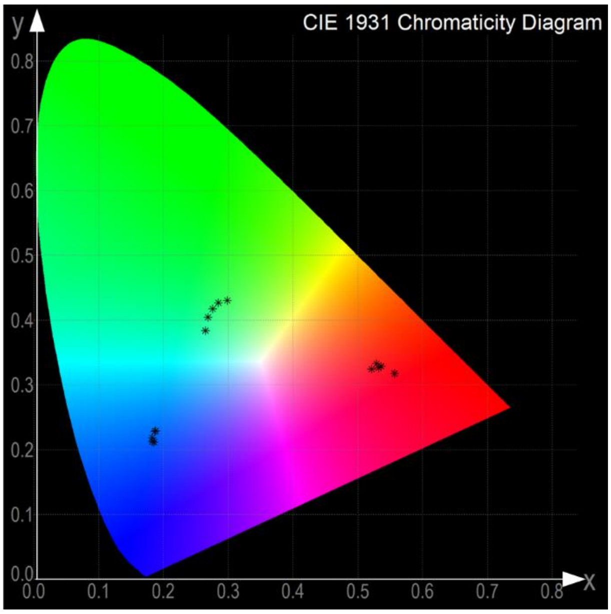

Figure S4 | The CIE 1931 chromaticity coordinates of the three primary color filters for the unpolarized light at the incidence angles of 0°,15°,30°,40°,50° corresponding to the measured results in Figure 2(a-c).

On the basis of these measured reflectance spectrums, the chromaticity coordinates at various angles of incidence are calculated and marked in Figure S4. A standard illuminant E is adopted in the color difference calculation, which has constant spectral power distributions over the whole visible spectrum. Though the chromaticity coordinates make a small movement with angle of incidence, the reflected specular color makes little change for all the fabricated RGB devices. Compared with the green color filter, the blue and red ones present a much higher saturation because of the efficient suppression at the blocking region and the narrower bandwidth.
